# On the Synergistic Effects of Cold Atmospheric Pressure Plasma Irradiation and Electroporation on Cytotoxicity of HeLa Cells

**DOI:** 10.3390/ijms26031093

**Published:** 2025-01-27

**Authors:** Nao Kitajima, Kosuke Makihara, Hirofumi Kurita

**Affiliations:** Department of Applied Chemistry and Life Science, Toyohashi University of Technology, Toyohashi 441-8580, Aichi, Japan

**Keywords:** cold atmospheric pressure plasma, plasma medicine, reactive oxygen and nitrogen species, electroporation, pulsed electric field, membrane permeabilization

## Abstract

Cold atmospheric plasma (CAP) treatment induces cancer cell death through the generation of reactive oxygen and nitrogen species (RONS). However, the efficacy of RONS delivery into cells remains limited by membrane permeability. Here, we investigated whether combining CAP with pulsed electric fields (PEFs) could enhance cancer cell death through increased intracellular RONS uptake. HeLa cells were treated with argon atmospheric pressure plasma jet (Ar-APPJ), PEF, or their combination. The combined treatment showed significantly enhanced cell death compared to single treatments. While PEF treatment alone induced membrane permeabilization, the combination with Ar-APPJ resulted in more pronounced and sustained membrane disruption, as evidenced by increased calcein leakage. This enhanced effect was attributed to Ar-APPJ-induced lipid peroxidation interfering with membrane resealing after PEF-induced electroporation. We also demonstrated that PEF-induced membrane electroporation facilitates the intracellular uptake of CAP-generated RONS. These findings provide mechanistic insights into the synergistic effects of combined CAP and PEF treatments, suggesting enhanced cell death via multiple pathways.

## 1. Introduction

Cold atmospheric pressure plasma (CAP) has emerged as a promising technology across various biological applications, including cancer therapy [1,2,3,4,5], wound healing [6,7], dentistry [8,9], orthopedics [10,11], and sterilization [12]. In particular, CAP treatment has demonstrated selective cytotoxicity against cancer cells both in vitro and in vivo through the generation of reactive oxygen and nitrogen species (RONS). This selective effect is also observed with CAP-irradiated solutions, such as cell culture media [5,13]. Its application has expanded to agriculture, particularly in seed treatment, disease prevention, and the modification of soil [14,15]. These biological effects are primarily mediated by RONS, which trigger changes in biomolecular structures, gene expression, and cellular signaling pathways.

RONS generated by CAP play crucial roles in cellular signaling and fate determination. In cancer cells, CAP-generated RONS, particularly hydrogen peroxide (H_2_O_2_), trigger oxidative stress by overwhelming the antioxidant defense systems. This selective vulnerability of cancer cells to oxidative stress is partly due to their altered redox status and elevated basal RONS levels compared to normal cells [16]. The CAP-induced oxidative stress activates various cellular signaling pathways, including mitogen-activated protein kinase (MAPK) cascades and p53-dependent pathways, ultimately leading to cell cycle arrest and apoptosis [17]. Furthermore, long-lived RONS produced in CAP-treated solutions can maintain their biological activity for extended periods, enabling indirect plasma treatment approaches [5,13]. These RONS primarily enter cells through specific membrane channels and carriers, such as aquaporins (AQPs) for H_2_O_2_ transport, although passive diffusion also occurs to some extent [18,19]. The differential expression of these transport systems between cancer and normal cells may contribute to the selective effects of CAP treatment.

The application of pulsed electric fields (PEFs) temporarily disrupts cell membrane integrity, creating pores that increase membrane permeability. This phenomenon, known as electroporation or electropermeabilization, enables the delivery of cell-impermeable molecules into cells [20,21,22,23]. The formation of these membrane pores occurs when the applied electric field exceeds a critical threshold, causing local defects in the phospholipid bilayer structure [22]. These structural changes can be either reversible or irreversible, depending on the electric field strength and pulse parameters. While conventional PEFs with microsecond durations are widely used for gene transfection in vitro and in vivo, they have also been applied in electrochemotherapy for delivering anticancer agents into tumor cells [24,25]. The efficiency of molecular delivery through electroporation depends on various factors, including membrane composition, cell size, and the physicochemical properties of the target molecules. Recent advances have led to the development of nanosecond PEFs (nsPEFs). These ultra-short pulses can induce distinct cellular responses, including intracellular calcium release, mitochondrial perturbation, and apoptosis, without causing significant thermal damage [26,27]. The unique ability of nsPEFs to access intracellular structures has opened new possibilities for cellular manipulation and therapeutic applications.

Recent studies have explored potential synergistic effects of combining atmospheric pressure plasma jet (APPJ) irradiation with PEF exposure. Jiang et al. demonstrated synergistic effects of nanosecond pulsed plasma jets and PEF on both cancer cell inactivation in vitro and plasmid DNA delivery in vivo [28,29]. Wolff et al. also showed that this combined treatment significantly enhanced intracellular RONS accumulation and immunogenic cell death in leukemia cells [30]. Furthermore, Chung et al. investigated the combined effects of indirect CAP treatment using CAP-treated phosphate-buffered saline with microsecond PEFs [31].

Based on these previous studies and our understanding of both technologies, we hypothesized that PEF-induced membrane electroporation could enhance the intracellular uptake of CAP-generated long-lived RONS, particularly H_2_O_2_, from the CAP-irradiated liquid, thereby promoting cancer cell death. To test this hypothesis, we investigated the synergistic effects of combined APPJ and PEF treatment on HeLa cells and examined the underlying mechanisms by analyzing intracellular RONS levels, membrane integrity, and lipid peroxidation.

## 2. Results

### 2.1. Cell Viability

**Figure 1:**
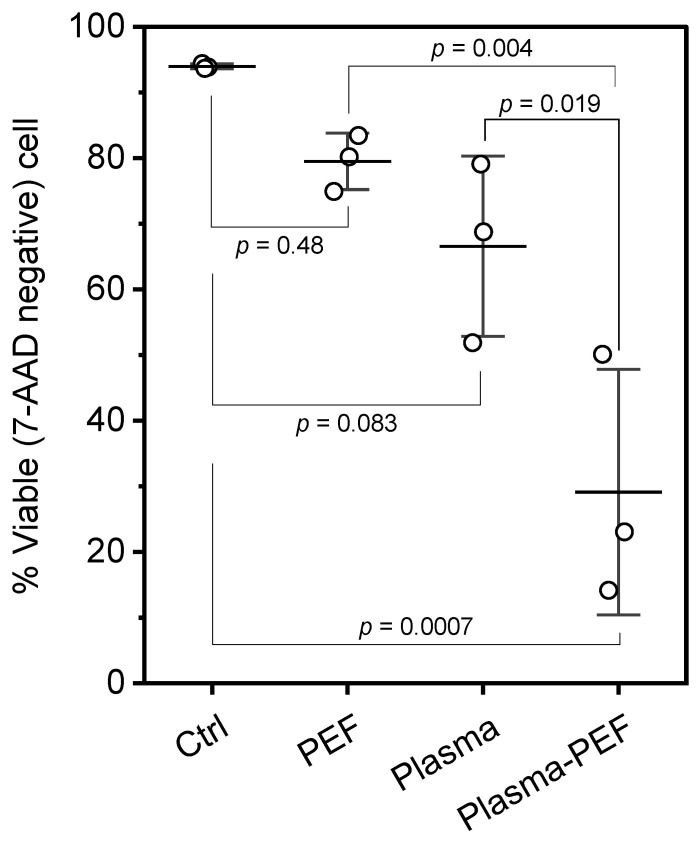
Cell viability 24 h after treatment measured using flow cytometry. HeLa cells were assayed according to 7-AAD uptake for cell death 24 h after treatment. Viability was calculated by dividing the number of 7-AAD-negative (viable) cells by the total number of cells. Data are expressed as the mean ± standard deviation (SD) (n=3). Statistical significance was determined using one-way ANOVA followed by Tukey’s multiple comparison tests.

Figure 1 shows cell viability 24 h after treatment. The treated HeLa cells were stained with the fluorescent dye 7-AAD and then analyzed by flow cytometry. The 7-AAD-negative cell was defined as a viable cell. 5 min of Ar-APPJ irradiation decreased the population of viable cells relative to the control (Ar gas flow for 5 min). PEF application also seemed to decrease cell viability, but there was no statistical difference between the control and PEF alone (p=0.48). The combination of Ar-APPJ and PEF resulted in a statistically significant decrease in cell viability compared to both the single application of Ar-APPJ and PEF. Therefore, our experimental condition has the synergistic effect of inducing cell death.

### 2.2. Intracellular RONS Level

**Figure 2:**
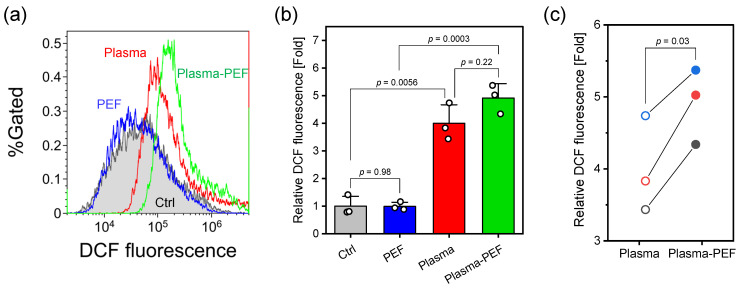
Intracellular RONS levels in HeLa cells treated with Ar-APPJ and PEF. (**a**) Representative flow cytometry histograms of DCF fluorescence. Control (Ctrl) represents cells treated with Ar gas flow for 5 min without plasma generation. (**b**) Relative median DCF fluorescence intensity normalized to control values. Data are expressed as the mean ± SD (n=3). Statistical significance was determined using one-way ANOVA followed by Tukey’s multiple comparison tests. (**c**) Changes in relative median DCF fluorescence intensity before and after PEF application. The same color indicates consecutive treatment. Statistical significance was determined using a paired *t*-test.

Flow cytometry histograms (Figure 2a) demonstrate that Ar-APPJ irradiation significantly increased 2′-7′dichlorofluorescein (DCF) fluorescence intensity compared with the control (Ar gas flow for 5 min), while PEF treatment alone showed no significant change. The combination of Ar-APPJ and PEF also enhanced DCF fluorescence intensity.

Quantitative analysis of relative median DCF fluorescence intensity normalized to control values is shown in Figure 2b. Both Ar-APPJ irradiation alone and the combination of Ar-APPJ and PEF significantly elevated the relative DCF fluorescence intensity. Although one-way ANOVA followed by Tukey’s multiple comparison tests showed no statistical difference between Ar-APPJ alone and the Ar-APPJ/PEF combination (p=0.22), these treatments represented paired conditions (Ar-APPJ with or without subsequent PEF application). Therefore, a paired *t*-test was performed to directly compare these specific groups (Figure 2c). The paired analysis revealed that PEF application following Ar-APPJ irradiation further increased the relative DCF fluorescence intensity. This analysis revealed that PEF application following Ar-APPJ irradiation significantly enhanced the relative DCF fluorescence intensity. As DCF fluorescence intensity correlates with intracellular RONS levels, these results indicate that Ar-APPJ irradiation elevated intracellular RONS concentration, and this effect was further enhanced by subsequent PEF treatment.

### 2.3. Membrane Integrity

**Figure 3:**
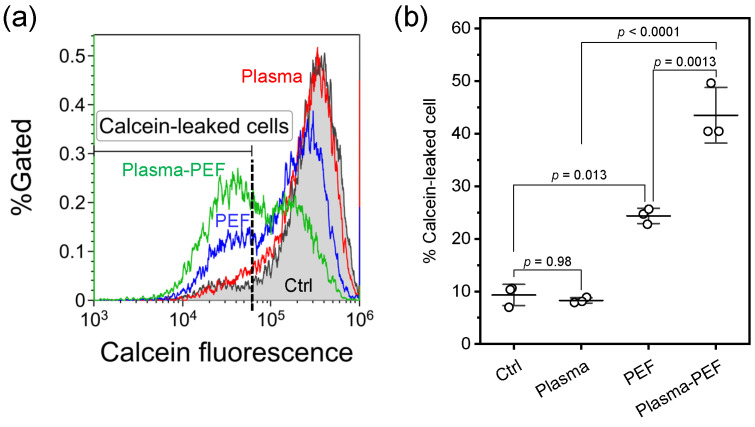
Membrane permeabilization assessed by calcein leakage in HeLa cells. (**a**) Representative flow cytometry histograms of calcein fluorescence. Control (Ctrl) indicates cells treated with Ar gas flow for 5 min without plasma generation. The dashed line represents the threshold for identifying cells with calcein leakage. (**b**) Percentage of cells showing calcein leakage. Data are expressed as the mean ± SD (n=3). Statistical significance was determined using one-way ANOVA followed by Tukey’s multiple comparison tests.

Figure 3 shows the results of the calcein leakage. Figure 3a shows typical flow cytometry histograms immediately after treatment. PEF applications increased the calcein-leaked cells; however, Ar-APPJ irradiation did not increase the calcein-leaked cells. A remarkable increase in the calcein-leaked cells was observed after the combination of Ar-APPJ and PEF. Figure 3b shows the population of the calcein-leaked cells. PEF applications increased the population of calcein-leaked cells; however, no statistically significant difference was observed between the control (Ar gas flow for 5 min) and Ar-APPJ. This result suggests that PEF generated calcein-permeable membrane pores, but Ar-APPJ irradiation did not. Moreover, a statistically significant increase in the population of the calcein-leaked cells was observed after the combination of Ar-APPJ and PEF treatment. Therefore, this result suggests that Ar-APPJ irradiation enhanced the pore formation stimulated by the following PEF applications.

### 2.4. Lipid Peroxidation

**Figure 4:**
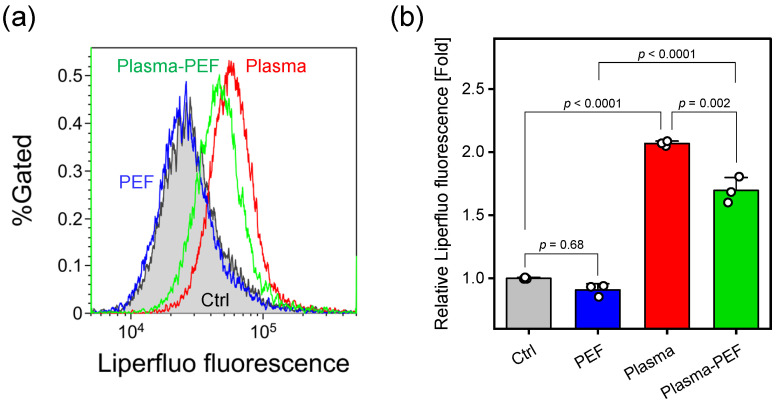
Assessment of lipid peroxidation in HeLa cells treated with Ar-APPJ and PEF. (**a**) Representative flow cytometry histograms of Liperfluo fluorescence. Control (Ctrl) indicates cells treated with Ar gas flow for 5 min without plasma generation. (**b**) Relative median Liperfluo fluorescence intensity normalized to control values. Data are expressed as the mean ± SD (n=3). Statistical significance was determined using one-way ANOVA followed by Tukey’s multiple comparison tests.

Figure 4 shows flow cytometry histograms (Figure 4a) and quantitative analysis of relative median Liperfluo fluorescence intensity normalized to control values (Figure 4b). While PEF treatment alone showed no significant change, Ar-APPJ irradiation significantly increased Liperfluo fluorescence intensity compared with the control (Ar gas flow for 5 min). The combination of Ar-APPJ and PEF also significantly elevated the relative Liperfluo fluorescence intensity compared to the control (p=0.0004), although the increase was less pronounced than with Ar-APPJ alone. Since Liperfluo fluorescence intensity correlates with the extent of lipid peroxidation, these results indicate that Ar-APPJ irradiation stimulated lipid peroxidation.

## 3. Discussion

In this study, we investigated the synergistic effect of cold atmospheric plasma (CAP) and pulsed electric fields (PEF) on HeLa cell death, focusing on the role of membrane electroporation in enhancing intracellular uptake of reactive oxygen and nitrogen species (RONS). We hypothesized that PEF-induced membrane electroporation enhances the intracellular uptake of long-lived RONS generated by CAP treatment in the CAP-irradiated liquid. To test this hypothesis, we examined cell viability, intracellular RONS levels, membrane integrity, and lipid peroxidation after treatment.

Cell viability was assessed 24 h after treatment (Figure 1). Single PEF application induced a moderate decrease in cell viability, which we attributed to membrane permeabilization. This membrane damage was later confirmed by calcein leakage assays (Figure 3), suggesting that the compromised membrane integrity led to the loss of essential cellular components such as ATP and ions. Similarly, Ar-APPJ treatment alone reduced cell viability, likely due to elevated intracellular RONS levels, leading to oxidative stress-induced cell death, as demonstrated in subsequent experiments (Figure 2). Each single treatment maintained cell viability above 50%, providing suitable conditions for investigating potential synergistic effects. Initial experiments with more intense conditions caused excessive cell death, making it difficult to investigate the synergistic effects. Therefore, we selected the current experimental conditions to achieve moderate effects with each single treatment, allowing us to clearly observe the enhancement by the combination treatment. The combined Ar-APPJ and PEF treatment resulted in significantly lower cell viability compared to either single treatment, indicating a synergistic effect on cell death.

The impact of treatments on intracellular RONS levels was evaluated using the fluorescent probe DCF (Figure 2). Ar-APPJ treatment significantly elevated intracellular RONS levels compared to the control. While the complete mechanism of APPJ-induced intracellular RONS elevation remains under investigation, our previous work suggests that localized H_2_O_2_ accumulation in the target liquid during CAP treatment plays a crucial role [32]. Additionally, recent studies have demonstrated that AQPs facilitate the transmembrane diffusion of CAP-generated H_2_O_2_ [18,19], providing a potential mechanism for the increase in the intracellular RONS level.

Combined Ar-APPJ and PEF treatment initially appeared to enhance DCF fluorescence. However, membrane integrity studies using calcein revealed that PEF treatment induced significant probe leakage from cells. To distinguish between actual RONS elevation and artifacts from probe leakage, we developed a sequential protocol: CM-H_2_DCFDA-loaded HeLa cells were exposed to Ar-APPJ for 5 min, transferred to fresh culture medium to neutralize extracellular RONS, centrifuged (400× *g*, 5 min, 4 °C), resuspended in D-PBS (-), and then subjected to PEF treatment.

**Figure 5:**
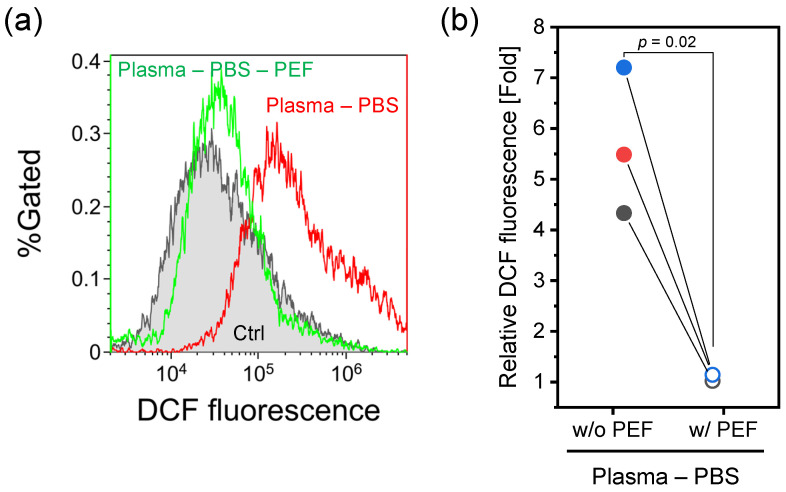
Sequential assessment of DCF retention in Ar-APPJ-treated HeLa cells before and after PEF application. (**a**) Representative flow cytometry histograms of DCF fluorescence. Control (Ctrl) represents cells treated with Ar gas flow for 5 min without plasma generation. “Plasma–PBS” indicates cells treated with Ar-APPJ, followed by medium exchange and resuspension in D-PBS (-). “Plasma–PBS–PEF” indicates cells subsequently treated with PEF after resuspension in D-PBS (-). (**b**) Changes in relative median DCF fluorescence intensity before and after PEF application. The same color indicates consecutive treatment. Statistical significance was determined using a paired *t*-test.

Flow cytometry analysis (Figure 5) revealed that DCF fluorescence intensity remained elevated after Ar-APPJ irradiation and buffer exchange compared to control levels. This observation confirmed intracellular accumulation of RONS and retention of the probe within cells prior to PEF application. However, subsequent PEF treatment decreased DCF fluorescence intensity to control levels, indicating that PEF induced probe efflux similar to that observed with calcein. These results suggest that the increased DCF fluorescence observed with combined Ar-APPJ and PEF treatment reflects both increased intracellular RONS levels and probe efflux. Consequently, the actual elevation of intracellular RONS levels was likely underestimated in our measurements.

In combined Ar-APPJ and PEF treatment, the driving force for RONS permeation into cells during PEF treatment is the concentration gradient. Our previous study showed that APPJ irradiation generates several hundred micromolar of H_2_O_2_ in D-PBS (-) [33]. On the other hand, H_2_O_2_ concentrations in mammalian cells can range from 1 to 700 nM [34]. Therefore, after Ar-APPJ irradiation of the cell suspension, long-lived RONS remain present, and when membrane pores are formed by PEF, these long-lived RONS diffuse into the cells. The observed synergistic enhancement of cell death, combined with these mechanistic insights, supports our hypothesis that PEF-induced membrane electroporation facilitates the intracellular uptake of CAP-generated long-lived RONS, although probe leakage presents a technical limitation for precise quantification.

Membrane integrity studies using calcein leakage revealed significant membrane permeabilization effects of the treatments (Figure 3). While PEF alone significantly increased calcein leakage (p=0.013 compared to control), the combination of Ar-APPJ and PEF treatment showed an enhanced effect (p=0.0013 compared to PEF alone). This synergistic enhancement of membrane permeabilization may be attributed to multiple factors. First, Ar-APPJ-induced lipid peroxidation, confirmed by the presence of lipid hydroperoxides in treated cells (Figure 4), likely altered membrane fluidity and stability [35,36]. Our previous investigations showed that hydroxyl radicals and superoxide were produced during APPJ irradiation [33]. These short-lived and highly reactive species could induce lipid peroxidation [37]. Second, these structural changes in the membrane lipids may have interfered with the natural membrane resealing process that typically occurs after PEF-induced electroporation [35,36]. This mechanism could explain the enhanced calcein leakage observed in our experiments.

Computational studies have demonstrated that lipid oxidation reduces the energy barrier for pore formation during electroporation [38,39]. This molecular mechanism may explain our observation that Ar-APPJ-induced lipid peroxidation enhanced the membrane permeabilization effects of subsequent PEF treatment. Furthermore, these simulations revealed that oxidized lipids interfere with membrane resealing, supporting our hypothesis regarding the sustained membrane permeability observed in our experiments. Together with our experimental observations, these molecular insights demonstrate that the combination of oxidative stress and electroporation creates a more severe and persistent disruption of membrane integrity than either treatment alone.

Our approach demonstrates key differences from previous studies by Jiang et al. [28,29] and Wolff et al. [30], which utilized nsPEF. While these studies showed synergistic effects, they did not specifically focus on membrane integrity assessment. In contrast, our application of millisecond pulse width PEF generates larger membrane pores, leading to more significant impacts on membrane permeability and consequent cell death. Although Chung et al. [31] examined membrane electropermeabilization using indirect CAP treatment with CAP-treated phosphate-buffered saline combined with microsecond PEFs, our study employs direct Ar-APPJ irradiation to cell suspension. This fundamental methodological difference provides insight into the enhanced synergistic effects observed in our combined CAP treatment approach.

In this study, we focused on elucidating the molecular mechanisms underlying the enhanced cell death induced by the combination of Ar-APPJ and PEF, using HeLa cells as a model system. While our findings provide insights into the synergistic effects of these treatments, further studies using multiple cell lines and investigating the differences in cellular responses between normal and cancer cells will be necessary to discuss the therapeutic implications.

## 4. Materials and Methods

### 4.1. Cell Culture

HeLa cells (RIKEN BioResource Research Center, Tsukuba, Japan) were maintained in Dulbecco’s modified Eagle’s medium (DMEM) with high glucose and phenol red (FUJIFILM Wako Pure Chemical, Osaka, Japan), 10% fetal bovine serum (FBS, Thermo Fisher Scientific, Waltham, MA, USA), 100 units/mL penicillin, and 100 μg/mL streptomycin (PS, FUJIFILM Wako Pure Chemical) at 37 °C, 5% CO_2_. When cells cultured in T75 flasks reached 50–70% confluence, they were trypsinized with 0.25% trypsin-EDTA (FUJIFILM Wako Pure Chemicals), harvested by centrifugation, and suspended in Dulbecco’s modified phosphate-buffered saline without magnesium chloride and calcium chloride (D-PBS (-), FUJIFILM Wako Pure Chemicals). The cell density was adjusted prior to experiments.

### 4.2. Ar-APPJ Generator and Plasma Irradiation Setup

**Figure 6:**
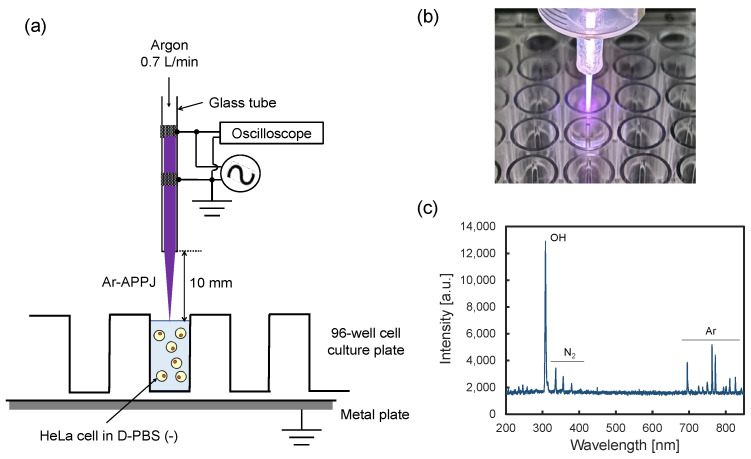
Ar-APPJ irradiation setup. (**a**) A schematic illustration of the Ar-APPJ irradiation setup. (**b**) A photograph of the Ar-APPJ. (**c**) Optical emission spectrum of Ar-APPJ. Characteristic emission lines of Ar (690–850 nm), N_2_ 2nd positive system (330–400 nm), and OH (306–309 nm) are shown [41].

Figure 6 shows the Ar-APPJ irradiation setup. The APPJ generator consisted of a quartz glass tube with an inner/outer diameter of 1.5/2.1 mm, with two copper tape electrodes (10 mm width) spaced 5 mm apart [33,40]. The upper electrode was powered, and the lower electrode was grounded. The glass tube was fixed in a plastic syringe (Terumo, Tokyo, Japan). The applied voltage (18 kV_p-p_ sinusoidal voltage at 18 kHz) was monitored by an oscilloscope (TDS2014, Tektronix, Beaverton, OR, USA) with a high-voltage probe (P6015A, Tektronix). The high-voltage generation system comprised a DC power supply (PK80H, Matsusada Precision, Kusatsu, Shiga, Japan) connected to a neon-sign inverter transformer (ALPHA NEON M-5, Lecip, Motosu, Gifu, Japan) that provided voltage step-up through high-frequency switching. The gap between the grounded electrode and nozzle was 10 mm. Argon (>99.99 vol% purity) served as the carrier gas at 0.7 L/min, regulated using a float-type flow meter (Kofloc, Nagoya, Aichi, Japan). Discharge power was determined to be less than 1 W using the Lissajous method. The optical emission spectrum shown in Figure 6c was acquired using a fiber-optic multichannel spectrometer (USB4000, Ocean Optics, Dunedin, FL, USA) under the identical operating conditions. The optical fiber probe was positioned 5 mm below the Ar-APPJ nozzle. Approximately 300 μL of a 1.0 × 10^6^ cells/mL cell suspension was added to one well of a 96-well cell culture plate. The gap between the nozzle and the surface of the cell suspension was fixed at 10 mm. The cell suspension was irradiated with the Ar-APPJ for 5 min at room temperature. No significant temperature change was observed compared to Ar gas flow without discharge. To compare the PEF single application to the combination treatment of APPJ first and PEF second, argon gas was irradiated to the cell suspension for 5 min. For the APPJ single application, 150 μL of the cell suspension was recovered in 6 mL of DMEM/10%FBS/PS and cultured for 24 h at 37 °C, 5% CO_2_.

### 4.3. PEF Application

PEFs were generated using a commercially available electroporator (NEPA21, Nepa Gene, Chiba, Japan). PEF was applied as two consecutive pulses. The PEF pulse duration was 5 ms, the pulse interval was 50 ms, the maximum electric field strength was 1.0 kV/cm, and the duty ratio was 10%. For the PEF treatment, the cell suspension following Ar-APPJ irradiation (less than 150 μL) was transferred into 2 mm-gap electroporation cuvettes (Nepa Gene). The time interval between Ar-APPJ and PEF treatments was kept at 1 min. After PEF application, the cell suspension was recovered in 6 mL of DMEM/10%FBS/PS and cultured for 24 h at 37 °C, 5% CO_2_.

### 4.4. Cell Viability

After the treatments, the cells were cultured in DMEM/10%FBS/PS for 24 h at 37 °C, 5% CO_2_. Cells contained in the medium or any washes and cells detached by trypsinization were harvested by centrifugation. Following resuspension in D-PBS (-), 7-amino-actinomycin D (7-AAD, Beckman Coulter, Brea, CA, USA) was added to the cell suspension to stain dead cells. Following incubation with 7-AAD for 15 min at room temperature, the fluorescence intensity of the cells was measured using flow cytometry.

### 4.5. Intracellular RONS Level

Intracellular RONS levels were determined using a general oxidative stress fluorescence probe 5-(and-6)-chloromethyl-2′,7′-dichlorodihydrofluorescein diacetate, acetyl ester (CM-H_2_DCFDA) (Thermo Fisher Scientific), as described in our previous papers [32,33,40]. Before the treatments, HeLa cells suspended in D-PBS (-) were incubated with 10 μM CM-H_2_DCFDA for 30 min at 37 °C. The probe-loaded cells were then centrifuged at 400× *g* for 5 min at 4 °C, resuspended in D-PBS (-), and adjusted to 1.0 × 10^6^ cell/mL. Approximately 300 μL of the cell suspension in one well of a 96-well cell culture plate was irradiated with the Ar-APPJ or argon gas for 5 min. After plasma irradiation of the cell suspension, the cell suspension was transferred into the electroporation cuvette. Following PEF application, the fluorescence intensity of the cells was measured using flow cytometry.

### 4.6. Membrane Integrity

Membrane integrity was monitored using a cell-impermeant fluorescent dye, calcein. This experiment was conducted as previously described [42,43] with some modifications. HeLa cells were incubated with 0.5 μM calcein-AM (Dojindo, Kumamoto, Japan) in D-PBS (-) for 30 min at 37 °C. After incubation, the cells were harvested by centrifugation and resuspended in D-PBS (-). Following the adjustment of cell concentration, the cell suspension was treated as described above. The fluorescence intensity of the cells was measured using flow cytometry.

### 4.7. Lipid Peroxidation

Lipid peroxidation was assessed by using Liperfluo reagent (Dojindo, Kumamoto, Japan) according to the manufacturer’s instruction manual. HeLa cells were treated with 5 μM Liperfluo for 30 min at 37 °C, 5% CO_2_. Cells were washed with D-PBS (-), detached by trypsinization, and resuspended in D-PBS (-), as described above. The cell suspension was treated as described above. The fluorescence intensity of the cells was measured using flow cytometry.

### 4.8. Flow Cytometry

The fluorescent intensity of the individual cells was measured using a CytoFLEX flow cytometer (Beckman Coulter) with data acquisition software CytExpert (Beckman Coulter). Before flow cytometry measurements, samples were filtered through a 35-μm nylon cell strainer (Corning, NY, USA) to remove cell debris. Data acquisition was performed on the cell size-corresponding population in the FSC/SSC plot, with a minimum of 10,000 events recorded for each experimental point. Kaluza Analysis 2.1 software (Beckman Coulter) was used for data analysis.

## 5. Conclusions

In conclusion, our study demonstrates a synergistic effect between Ar-APPJ and PEF treatments on HeLa cell death through multiple mechanisms. We have shown that PEF treatment induces significant membrane permeabilization, as evidenced by calcein leakage assays. The combination of Ar-APPJ and PEF resulted in enhanced membrane permeability beyond that observed with PEF alone, which we attribute to Ar-APPJ-induced lipid peroxidation. This was supported by the detection of lipid hydroperoxides in Ar-APPJ-treated cells, suggesting that oxidative damage to membrane lipids may impair membrane resealing after electroporation. While probe leakage presented technical limitations for precise quantification, our sequential treatment protocol provided evidence that PEF-induced membrane electroporation can facilitate the intracellular uptake of CAP-generated RONS. These findings provide new insights into the mechanisms underlying the synergistic effects of combined CAP and PEF treatments, suggesting potential applications in cancer therapy where enhanced cell death through multiple pathways is desired. Further studies investigating the detailed molecular mechanisms (particularly the role of specific RONS species and membrane repair pathways) and optimization of treatment parameters (such as pulse duration, field strength, and treatment timing) may lead to the development of more effective combinations.

## Data Availability

The original contributions presented in this study are included in the article. Further inquiries can be directed to the corresponding author.
